# Expanding the PARiHS framework: thinking more broadly about context and facilitation

**DOI:** 10.1186/1472-6963-14-S2-O10

**Published:** 2014-07-07

**Authors:** Robin Urquhart, Joan Sargeant, Geoffrey Porter, Lois Jackson, Eva Grunfeld

**Affiliations:** 1 Department of Surgery, Dalhousie University, Halifax, Nova Scotia, Canada; 2 Division of Medical Education, Dalhousie University, Halifax, Nova Scotia, Canada; 3 School of Health and Human Performance, Dalhousie University, Halifax, Nova Scotia, Canada; 4 Department of Family and Community Medicine, University of Toronto, Toronto, Ontario, Canada

## Background

The movement of innovations (i.e., new knowledge and tools) into healthcare settings is a significant challenge. The Promoting Action on Research Implementation in Health Services (PARiHS) framework proposes that the successful implementation of research into practice is a function of the interaction between three elements: 1) evidence; 2) context; and 3) facilitation. The objective of this paper is to present data from a study of innovation implementation in Nova Scotia, Canada, which extends and refines our understanding of PARiHS.

## Materials and methods

We used case study methodology to examine the multi-level factors influencing the implementation of synoptic reporting tools (SRTs) for mammography, endoscopy, and cancer surgery reporting. SRTs capture and present information about a medical or surgical procedure in a structured, checklist-like format and typically report only items critical for understanding the disease and subsequent impacts on patient care. Three theoretical perspectives, including PARiHS, were used as conceptual bases for the study. Data were collected through interviews with 55 key informants, document analysis, nonparticipant observation, and tool use/examination. Analysis included a thematic analysis of each case, which involved iterative processes linking case-specific data to the theoretical perspectives, and a cross-case analysis to compare and contrast the themes across cases.

## Results

PARiHS characterizes context using the sub-elements of culture, leadership, and evaluation. In this study, these specific sub-elements were influential in one case only. Additional features of context, however, had important influences on SRT implementation across the cases. These included: availability of specific organizational resources (e.g., time, expertise); structural, infrastructural, and regulatory components of the broader healthcare system; and the history and nature of both intra- and inter-organizational relationships. PARiHS defines facilitation primarily as a role that an individual (or “trained expert”) fills, and views facilitation on a continuum from low (task-focused; “doing for others”) to high (holistic; “enabling others”). In this study, rather than a role filled by a distinct individual, facilitation was demonstrated both as a set of activities deliberately employed by implementation teams to facilitate the implementation process and as a team or organizational capacity with many individuals (e.g., clinical champions, supportive middle managers/department heads) adopting facilitation roles. Finally, task-focused facilitation was critical to realizing implementation in all three cases.

## Conclusions

The findings suggest that PARiHS may present a relatively narrow view of context and facilitation. These findings provide a basis to build on new concepts and to expand our understanding of the elements of this framework.

